# *Curling Leaf 1*, Encoding a MYB-Domain Protein, Regulates Leaf Morphology and Affects Plant Yield in Rice

**DOI:** 10.3390/plants12173127

**Published:** 2023-08-31

**Authors:** Dandan Guo, Lianghai Chen, Shiqiang Liu, Wenxiang Jiang, Qing Ye, Zheng Wu, Xiaoqing Wang, Xiafei Hu, Zelin Zhang, Haohua He, Lifang Hu

**Affiliations:** 1 College of Agriculture, Jiangxi Agricultural University, Nanchang 330045, China; gddan2022@163.com (D.G.); jwx1744725022@163.com (W.J.); yeqing19821238532@163.com (Q.Y.); wz2022zc@163.com (Z.W.); wangxiaoq2022@163.com (X.W.); hxf4394016@163.com (X.H.); zzl2296938807@163.com (Z.Z.); 2 Key Laboratory of Crop Physiology, Ecology and Genetic Breeding, Ministry of Education, Jiangxi Agricultural University, Nanchang 330045, China; 15870644120@163.com (L.C.); lsq_hn306@163.com (S.L.); 3 College of Bioscience and Bioengineering, Jiangxi Agricultural University, Nanchang 330045, China

**Keywords:** rice, *CL1*, curling leaf, yield

## Abstract

The leaf is the main site of photosynthesis and is an important component in shaping the ideal rice plant architecture. Research on leaf morphology and development will lay the foundation for high-yield rice breeding. In this study, we isolated and identified a novel curling leaf mutant, designated *curling leaf 1* (*cl1*). The *cl1* mutant exhibited an inward curling phenotype because of the defective development of sclerenchymatous cells on the abaxial side. Meanwhile, the *cl1* mutant showed significant reductions in grain yield and thousand-grain weight due to abnormal leaf development. Through map-based cloning, we identified the *CL1* gene, which encodes a MYB transcription factor that is highly expressed in leaves. Subcellular localization studies confirmed its typical nuclear localization. Transcriptome analysis revealed a significant differential expression of the genes involved in photosynthesis, leaf morphology, yield formation, and hormone metabolism in the *cl1* mutant. Yeast two-hybrid assays demonstrated that CL1 interacts with alpha-tubulin protein SRS5 and AP2/ERF protein MFS. These findings provide theoretical foundations for further elucidating the mechanisms of *CL1* in regulating leaf morphology and offer genetic resources for practical applications in high-yield rice breeding.

## 1. Introduction

Rice (*Oryza sativa* L.) is one of the most important staple crops in the world. Unraveling the genetic mechanisms underlying rice yield formation and developing new high-yielding rice varieties are crucial for ensuring national food security. Leaves, as the vital organs responsible for photosynthesis and gas exchange, play a determinant role in yield formation [1]. Therefore, leaf morphological improvement has long been recognized as a key objective in plant architecture enhancement for rice breeding programs [2]. The identification and cloning of genes related to rice leaf morphogenesis can contribute to elucidating the genetic regulatory mechanisms underlying rice plant architecture. Furthermore, these findings can serve as genetic resources for high-yield rice breeding when applied in practice.

The development of rice leaves is a complex process that initiates from the peripheral region of the shoot apical meristem (SAM). The primary meristematic cells in this region differentiate into leaf primordia and subsequently undergo differentiation along the adaxial–abaxial axis (polar development along this axis, with the adaxial side facing the stem and the abaxial side facing away from the stem), determining the three-dimensional spatial morphology of rice leaves [3]. A number of leaf mutants displaying abnormal leaf width and curling have been identified in rice, with the curling phenotype further classified into inward curling (adaxial) and outward curling (abaxial) [4]. The curling of rice leaves is primarily influenced by physiological processes such as osmotic pressure changes, drought, and hormones, as well as the intrinsic genes involved in polarity establishment, cell differentiation, and regulation by transcriptomic microRNAs [5]. Among these factors, polarity establishment plays a particularly important role in the formation of curled leaves in rice and is mainly regulated by sclerenchymatous cells and bubble cells [6].

Several isolated genes, such as *SRL2* [7,8], *OsAGO1b* [9], *RLM3* [10], and *NRL3* [11], can affect the degree of leaf curl by regulating the development of distal sclerenchymatous cells.

*SRL2* regulates the development of sclerenchymatous cells on the abaxial side; the abnormal development of these cells leads to changes in leaf polarity and subsequent leaf curling [7,8]. The overexpression of *OsAGO1b* induces leaf curling toward the adaxial side by affecting the development of sclerenchymatous cells at the distal end [9]. *RLM3* regulates the development of secondary cell walls in rice, thereby influencing leaf curling [10]. *NRL3* regulates the development of sclerenchymatous cells and vascular bundle numbers, resulting in narrower leaf curling [11].

Genes such as *REL1* [12,13], *URL1* [14], *ACL1* [15], and *PSL1* [16] have been attributed to regulating leaf curling by controlling the development of bubble cells at the adaxial axis. In the rel1 mutant, there is an increase in the size and number of bubble cells in the leaves, accompanied by alterations in the contours of these cells, resulting in leaf curling [12,13]. *URL1* is involved in leaf curling due to variations in the number and size of bubble cells [14]. The *ACL1* gene regulates leaf curling through transcriptional repression, mediated by the *URL1-ROC5-TPL2* repressive complex [15]. *PSL1* can alter the cell wall structure in rice by encoding a galacturonate reductase, thereby inducing leaf curling [16].

It is noteworthy that many genes involved in regulating rice leaf development also influence seed size and grain yield. *IPA1* regulates the architecture of rice plants, inflorescence morphology, and leaf photosynthetic characteristics [17,18]. *NAL4* has an impact on leaf width and grain yield in rice [19,20,21]. The upregulation of *GL7* results in narrower leaves, while *GS2* affects both larger grain size and increased leaf length [22,23]. Considering the dual role of these genes in regulating leaf development and grain yield, they serve as important genetic resources for high-yield rice breeding. By using molecular genetic breeding techniques to modulate leaf morphology, improve light utilization efficiency, and balance the relationship between grain yield and leaf characteristics, it is possible to cultivate ideal plant varieties with enhanced productivity. This approach offers a promising means to achieve high-yield rice production effectively.

In this study, we isolated a gene, *Curling Leaf1* (*CL1*), which regulates rice leaf development and encodes a MYB protein. *CL1* was found to modulate leaf curling by participating in the development of sclerenchymatous cells on the abaxial side. Moreover, it also influenced grain development and yield. *CL1* likely serves as an important genetic resource for the cultivation of ideal plant architecture and high-yield breeding in rice.

## 2. Results

### 2.1. The *cl1* Mutant Is Characterized by the Loss of Sclerenchymatous Cells, Resulting in Inward Leaf Curling

**Figure 1:**
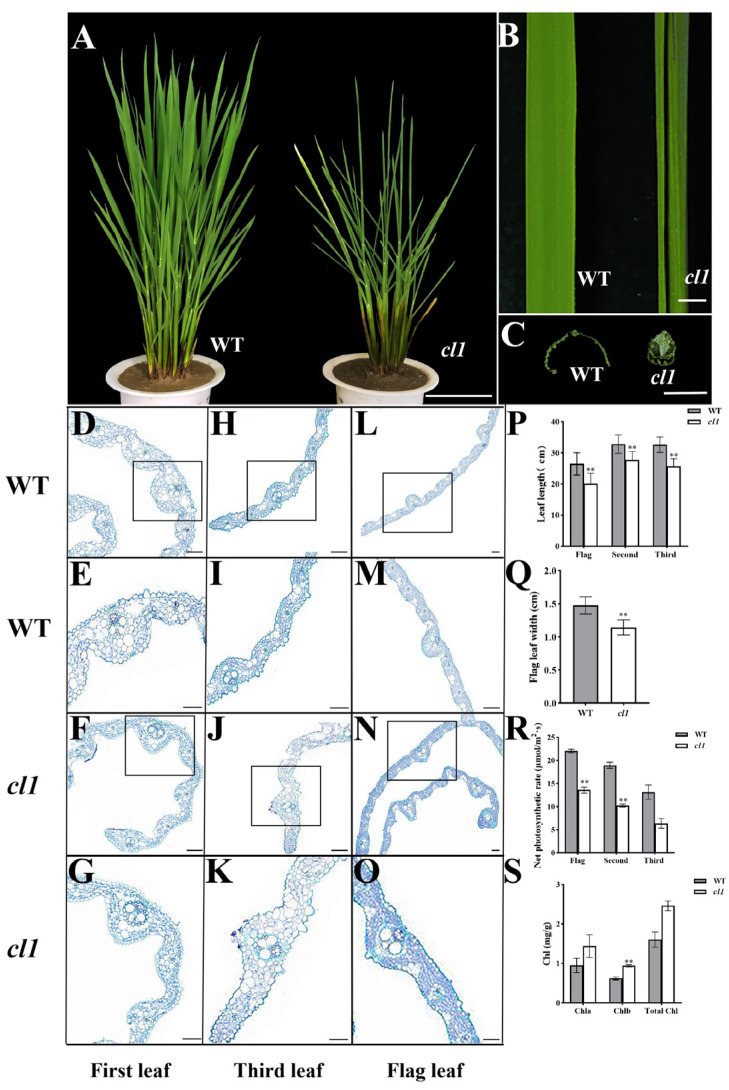
Comparison of the phenotypes of WT and *cl1* mutant plants. (**A**): Morphology of WT and *cl1* mutant plants at the tillering stage. (**B**): Phenotypes of the flag leaves in the WT and *cl1* mutant plants at the seedling stage. (**C**): The clear cells of the midrib in the WT and *cl1* mutant at the seedling stage. Bars = 10 cm (**A**), 0.5 cm (**B**) and 0.2 cm (**C**). (**D**–**O**): *cl1* leaf blades display altered cellular organization compared with WT. The cross-sectional morphology of the first leaf (**D**–**G**), third leaf (**H**–**K**), and flag leaf (**L**–**O**) in both WT and *cl1* mutant plants. (**D**,**E**,**H**,**I**,**L**,**M**) represent the WT, (**F**,**G**,**J**,**K**,**N**,**O**) represent the *cl1* mutant. (**E**,**G**,**I**,**K**,**M**,**O**) represent the enlarged portion in (**D**,**F**,**H**,**J**,**L**,**N**), respectively. Bars = 50 μm. (**P**–**S**): Leaf length (**P**), leaf width (**Q**), net photosynthetic rate (**R**), and chlorophyll content (**S**) in the WT and *cl1* mutant. The values indicate the means of three biological replicates ± SE. **: significant difference at *p* < 0.01 via *t*-test.

To isolate the novel genes regulating rice leaf development, we screened a curling leaf mutant, *curling leaf 1* (*cl1*), from a mutant library generated by ^60^Co-γ radiation of the WuYunJing 7 cultivar. Compared to the wild type (WT), the *cl1* mutant exhibited slight inward leaf curling, starting from the first leaf, with the degree of curling increasing with leaf age until complete curling is achieved, the leaf then resembling a scallion shape (Figure 1A–O). Histological analysis revealed significant changes in the differentiation and distribution of mesophyll cells in the *cl1* mutant. In the wild-type leaves, the adaxial side of the minor veins consisted of sclerenchymatous cells (Figure 1D–I), whereas in the *cl1* mutant, the sclerenchymatous cells were absent on the adaxial side of the minor veins and were replaced by mesophyll cells (Figure 1J–O). Additionally, the polarity of the *cl1* mutant leaves was visibly altered, with bubble cells localized on the abaxial side instead of the median adaxial side, as observed in the WT. Furthermore, the degree of leaf curling in the *cl1* mutant was greater than that in wild-type leaves of the same leaf age (Figure 1A–O). These results indicate that the *cl1* mutant is characterized by the defective development of sclerenchymatous cells at the distal end, leading to altered distribution of mesophyll cells and subsequent inward leaf curling. In terms of leaf color, the *cl1* mutant displayed a darker shade of green than the WT (Figure 1A–C). Additionally, the leaf lengths of the flag leaf, second leaf, and third leaf were significantly reduced in the *cl1* mutant (Figure 1P), while the leaf width of the flag leaf was noticeably narrower (Figure 1S). Analysis of the relevant photosynthetic characteristics revealed a significant increase in chlorophyll b content but a significant decrease in the net photosynthetic rate in the *cl1* mutant. Hybridization of the *cl1* mutant with the WT resulted in F1 plants that exhibited normal leaf morphology. In the F2 segregating population, a typical 3:1 segregation ratio was observed between normal plants (372 individuals) and curling leaf plants (126 individuals), indicating that the mutant phenotype is controlled by a single recessive nuclear gene.

### 2.2. The *cl1* Mutant Exhibited a Significant Reduction in 1000-Grain Weight and a Consequent Decrease in Yield

**Figure 2:**
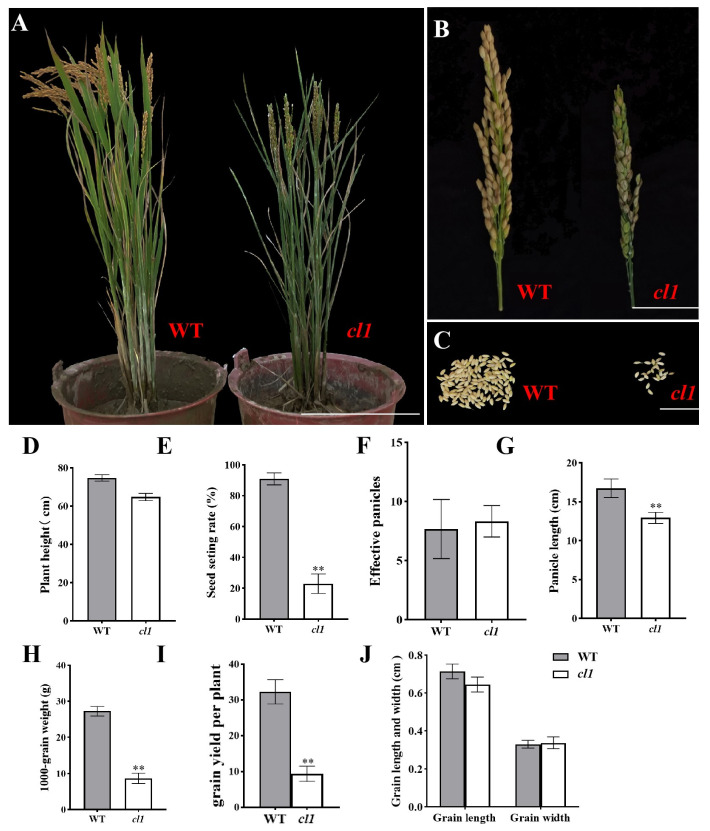
Comparison of the yield morphological characteristics in WT and *cl1* mutant plants. (**A**): WT (left) and *cl1* mutant (right) plants at the maturated stage. (**B**): WT (left) and *cl1* mutant (right) panicles. (**C**): WT (left) and *cl1* mutant (right) single spike yield. Bars = 20 cm (**A**), 0.2 cm (**B**), and 2 cm (**C**); (**D**–**J**): plant height (**D**), seed setting rate (**E**), effective panicles (**F**), panicle length (**G**), 1000-grain weight (**H**), grain yield per plant (**I**), and grain length and width (**J**). The values indicate the means of three biological replicates ± SEs. **: significant difference at *p* < 0.01 via *t*-test.

Agronomic trait analysis revealed that compared to the WT, the *cl1* mutant plants exhibited a shorter stature (Figure 2A,D) and an increased number of effective panicles (Figure 2F). However, the single spike yield (Figure 2B,C), panicle length (Figure 2C,G), and seed setting rate (Figure 2E) were significantly reduced in the *cl1* mutant, with its grain filling rate and 1000-grain weight accounting for only 25.3% and 31.9% of the WT, respectively (Figure 2H); the yield per plant decreased significantly (Figure 2I), with no significant difference in grain length and grain width (Figure 2J).

### 2.3. CL1 Encodes an MYB Transcription Factor

**Figure 3:**
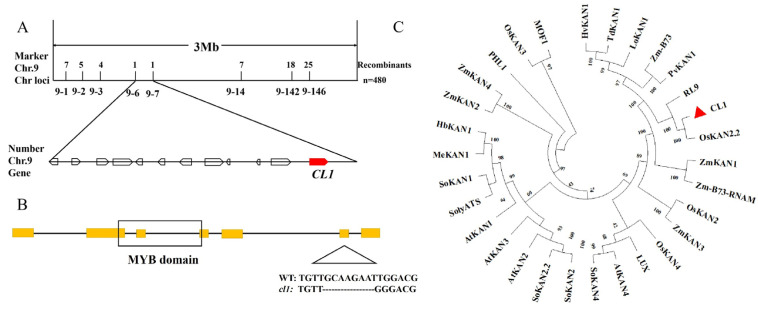
Map-based cloning and the evolutionary tree of *CL1*. (**A**): Mapping of *CL1*. *CL1* was mapped primarily to a 24.6 kb region on chromosome 9 between markers 9-6 and 9-7 in an F2 mapping population. (**B**): *CL1* (LOC_Os09g23200) encodes an MYB transcription factor. There were 8 bases (GCAAGAAT) missing at the exon 6 splice point in the *cl1* mutant, leading to premature translation termination. (**C**): Phylogenetic relationships analysis of CL1 proteins and the 30 homologs from eleven different species. The numbers at each node represent the bootstrap support (percentage). The scale bar indicates genetic distance, based on branch length. Note: The yellow region represents the exons of *CL1*, and the black box is the MYB domain.

To isolate the *CL1* gene, we used 480 segregants from the F2 populations and a set of polymorphic markers covering the entire genome, then mapped the mutant locus to a 3 Mb region on chromosome 9 between 9-6 and 9-7 (Figure 3A). Genome sequencing analysis between the WT and *cl1* mutant revealed that there was only one homozygous heterotopic point in the mapping region (Table S1 in the Supplementary Materials). The mutated gene is *LOC_Os09g23200*, which encodes an MYB transcription factor. *LOC_Os09g23200* was considered as a candidate gene for *CL1*. Sequencing and comparative analysis demonstrated the occurrence of missing 8 bases (GCAAGAAT) at the exon 6 splice point of *LOC_Os09g23200* in the *cl1* mutant (Figure 3B).

*LOC_Os09g23200* was characterized as having seven exons and six introns and encoding a 532 amino-acid protein with a putative MYB domain that belongs to the KANADI family. The mutation in the *cl1* mutant caused a frame shift of the protein and led to premature translation termination.

To determine the evolutionary relationship of *CL1* with its homologs from other species, we used BlastP with the *CL1* full-length amino acid sequence to find orthologs and create a phylogenetic tree. A total of 30 orthologous sequences were retrieved from eleven different species (*Oryza sativa* L., *Zea mays* L., *Arabidopsis thaliana* (L.) *Heynh.*, *Solanum lycopersicum* L., etc.). *CL1* contained a highly homologous sequence (more than 70% identity) with KANADI family proteins from other species in its KAN domain and had the highest homology with *OsKAN2.2* (Figure 3C).

### 2.4. The Expression Pattern and Subcellular Localization of CL1

**Figure 4:**
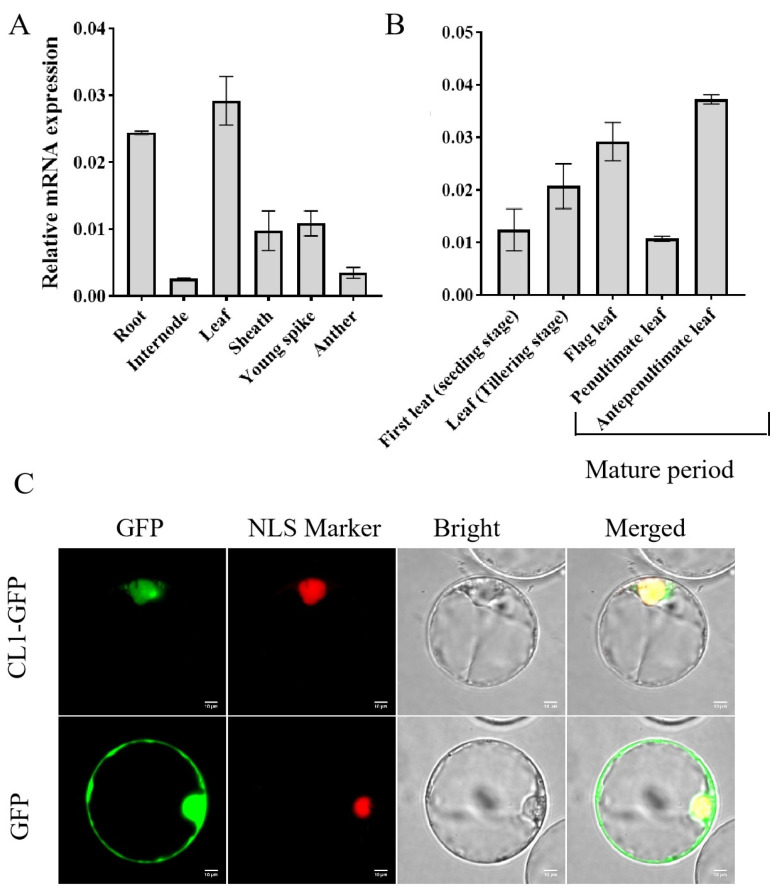
Real-time PCR expression analysis of *CL1*. (**A**): Real-time PCR analysis of *CL1* transcript levels in different organs; total RNA was isolated from rice roots (tillering stage), culms of the second internodes, young spikes, flag leaves, flag sheaths, and anthers. The values indicate the means of three biological replicates ± SEs. (**B**): Real-time PCR analysis of *CL1* transcript levels in different leaves; total RNA was isolated from rice from the first leaf, young leaf (tillering stage), flag leaf, penultimate leaf, and antepenultimate leaf. The values indicate the means of three biological replicates ± SEs. (**C**): Transient co-expression of *CL1*-GFP fusion protein and a nucleus marker in rice protoplasts revealed that *CL1* is mainly located in the nucleus. Bars = 10 µm.

Quantitative real-time RT–PCR (qRT–PCR) analysis was conducted to investigate the expression pattern of *CL1* across various tissues, including the roots, stems, leaves, young panicles, and anthers. The results revealed significant expression signals of *CL1* in all the tested tissues, with relatively higher expression levels observed in the leaves and roots (Figure 4A). In order to further understand the expression of *CL1* in the leaves, we extracted the RNAs of the first leaf at seedling stage, a leaf at the tillering stage, a flag leaf, the penultimate leaf, and the antepenultimate leaf at maturity stages for further analysis. It was found that *CL1* exhibited an expression throughout the different leaf developmental stages, with its expression level increasing as the leaves matured (Figure 4B).

To determine the subcellular localization of the *CL1* protein, a *CL1*-GFP fusion construct was generated and transformed into rice protoplasts for localization analysis. As shown in Figure 4C, the green fluorescence of *CL1* merged with the red fluorescence of the nuclear marker, indicating that *CL1* is predominantly localized in the nucleus. This observation confirms that *CL1* functions as a typical nuclear protein. These findings provide valuable insights into the expression pattern and subcellular localization of *CL1*, contributing to a better understanding of its functional roles in rice leaf development and the related processes.

### 2.5. Transcriptome Analysis of the cl1 Mutant

**Figure 5:**
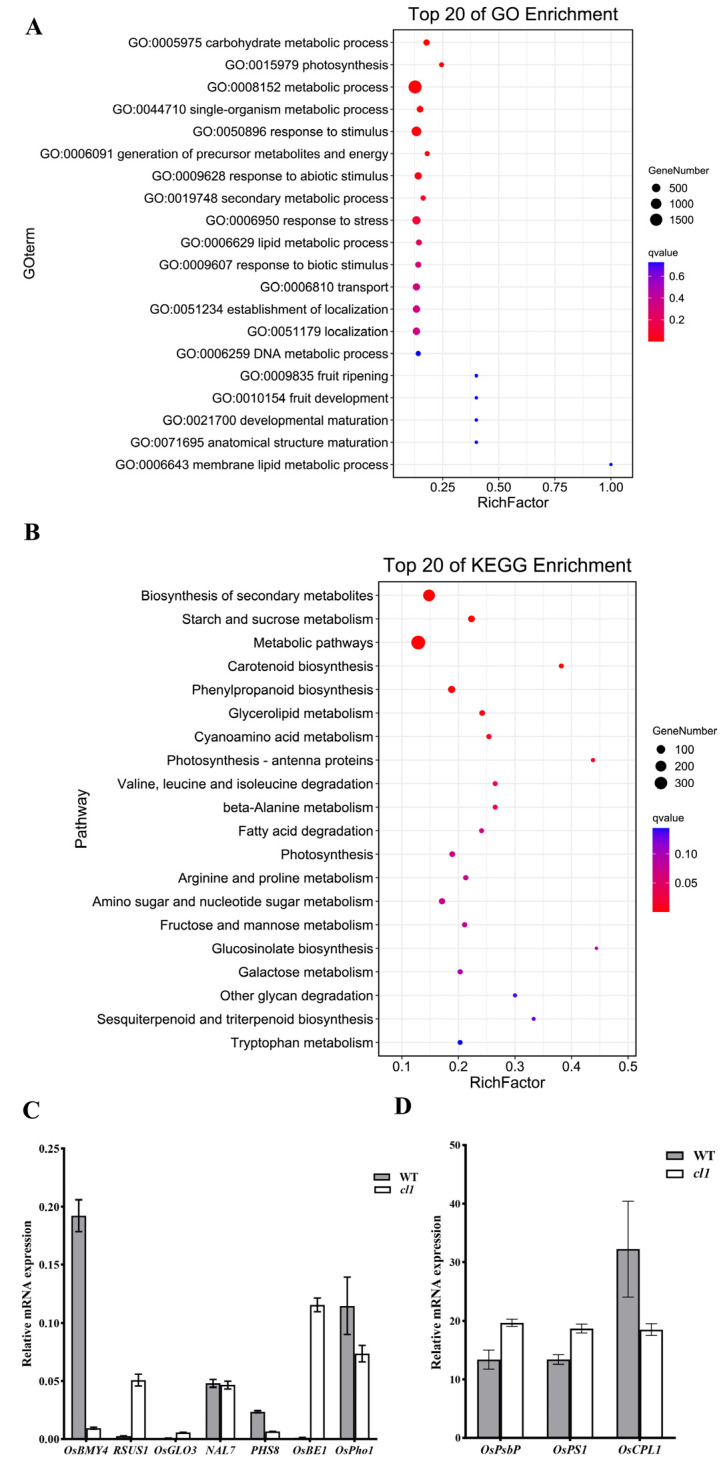
Transcriptome analysis of *cl1* mutant. (**A**): GO analysis of DEGs; (**B**): KEGG pathway enrichment analysis of DEGs. (**C**,**D**) qRT–PCR analysis of the differentially expressed genes related to leaf shape and yield regulation in the *cl1* mutant. The rice *Actin1* gene was used as a control. The values indicate the means of three biological replicates ± sEs.

In order to further elucidate the potential molecular regulatory mechanisms of *CL1* in controlling rice leaf morphology and yield, we selected the WT and *cl1* mutant leaves at the seedling stage for transcriptome analysis. Three randomly selected plants represented a biological duplication, with a total of three replicates. Using a filtering criterion of FDR < 0.05 and a fold change greater than two, a total of 3959 differentially expressed genes (DEGs) were identified, including 2413 upregulated genes and 1546 downregulated genes. To validate the accuracy of the transcriptomic sequencing results, ten genes involved in leaf development regulation (*OsBE1* [24], *OsPS1* [25], *OsPsbP* [26], *OsCPL1* [27], and *NAL7* [28]) and plant yield (*OsPho1* [29], *OsGLO3* [30], *OsBMY4* [31], *PHS8* [32], and *RSUS1* [33]) were selected for qRT–PCR analysis (Figure 5C,D). The results showed consistent expression trends between the ten DEGs and the corresponding RNA-Seq data, indicating the reliability of the data and its ability to reflect the transcript levels of genes in both WT and *cl1* mutant leaves. These findings provide a solid foundation for further analysis and investigation.

Through GO analysis, a total of 3959 DEGs were functionally annotated and classified into three major functional categories: molecular function, cellular component, and biological process. Within the molecular function category, these DEGs were further categorized into 15 subcategories, with the highest proportion observed in the catalytic activity subcategory (836 upregulated genes, 527 downregulated genes). In the biological process category, the DEGs were classified into 10 subcategories, with the highest proportions found in photosynthesis and the carbohydrate metabolic process (495 upregulated genes, 171 downregulated genes). In terms of the cellular component category, the DEGs were divided into five subcategories, with the highest proportions observed in the thylakoid and plastids (164 upregulated genes, 67 downregulated genes (Figure 5A).

To gain further insights into the metabolic regulatory networks in which the differentially expressed genes (DEGs) may be involved, KEGG annotation analysis was conducted on the DEGs. The analysis revealed that a total of 980 DEGs were associated with 116 metabolic pathways. By applying the significance threshold of a *p*-value (q < 0.05), 20 pathways were found to be significantly enriched (Figure 5B). Among them, the metabolic pathways phenylpropanoid biosynthesis and secondary metabolite biosynthesis exhibited the highest enrichment, with 464 upregulated genes and 240 downregulated genes. Within the significantly enriched pathways, several pathways related to photosynthesis, chlorophyll synthesis, and morphological regulation were identified, including photosynthesis-antenna proteins, carotenoid biosynthesis, and phenylpropanoid biosynthesis. Pathways associated with yield formation included starch and sucrose metabolism, beta-alanine metabolism, glycerolipid metabolism, and cyanoamino acid metabolism (128 upregulated genes and 45 downregulated genes). These findings provide valuable information regarding the potential involvement of DEGs in the metabolic pathways associated with photosynthesis, chlorophyll synthesis, morphological regulation, and yield formation. Considering the significant reduction in leaf curling, thousand-grain weight, and yield in the *cl1* mutant, we analyzed the expression levels of the relevant genes involved in rice leaf morphogenesis and yield regulation among the differentially expressed genes. The study revealed that among the differentially expressed genes, a total of 21 known leaf development genes were identified (Table S2 in the Supplementary Materials). Among them, the expression levels of five genes (*OsGPT1* [34], *OsMYB30* [35], *OsClpP5* [36], *OsBE1* [24], and *PSL1* [25]) showed a significant decrease, with downregulation fold changes ranging from 2.22 to 9.59. Conversely, the expression levels of the remaining 16 genes (including *OsTIC62* [37], *OsPORB* [38], *OscpSRP43* [39], *OsPsbP* [26], etc.) exhibited a significant increase, with upregulation fold changes ranging from 2.01 to 7.0. Furthermore, the differentially expressed genes also included 14 genes involved in yield regulation, out of which 8 genes (*PHS8* [32], *OsPho1* [29], *OsBMY4* [31], *OsAGPL1* [40], *OsSAPK9* [41], *OsSSI* [42], *OsAGPS1* [43], and *OsGlyI* [44]) showed a significant decrease in expression, with downregulation fold changes ranging from 2.00 to 9.18. Conversely, the expression levels of the remaining six genes (*CAP1* [45], *RSUS1* [33], *OsSSIIIa* [46], *OsFRK3* [47], *RSUS2* [48], and *OsF2KP1* [49]) exhibited a significant increase, with upregulation fold changes ranging from 2.05 to 4.25.

The aforementioned results Indicate that the *CL1* gene is a crucial factor in influencing rice leaf morphology, chloroplast development, the photosynthetic system, and yield composition. It is likely that *CL1* regulates photosynthesis and rice plant yield through the modulation of pathways such as ko00500 (involving genes such as *OsPho1* [29], *PHS8* [32], *OsBMY4* [31], and *RSUS1* [33]) and ko00195 (involving genes such as *OsPsbP* [26], *OsCPL1* [27], and *OsPS1* [25]). Furthermore, *CL1* may impact rice leaf morphology by influencing the expression of the genes involved in chlorophyll, chloroplast, and leaf mesophyll cell development.

### 2.6. CL1 Interacts with SRS5 and MFS

**Figure 6:**
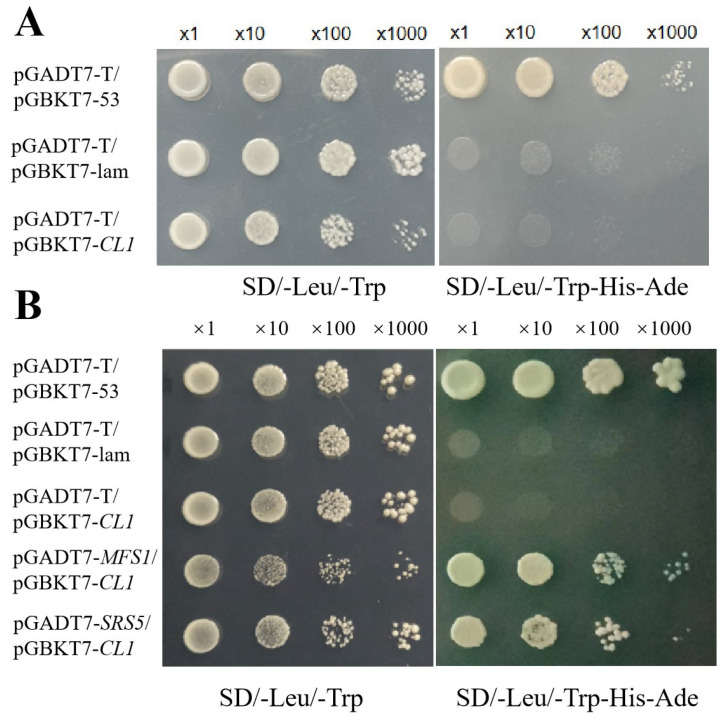
CL1 interacts with SRS5 and MFS. (**A**): Transcriptional activation assay of CL1 in yeast. The interaction between pGBKT-53 and pGADT7-T was used as the positive control; pGBKT7-lam + pGADT7-T was used as the negative control. (**B**): Yeast two-hybrid assays for detecting the interaction between CL1 with SRS5 and MFS. The interactions between pGBKT7-lam + pGADT7-T and pGBKT7-CL1 + pGADT7 were used as the negative control.

To further explore the molecular mechanisms of *CL1* in rice leaf morphogenesis and yield regulation, we constructed a rice whole-tissue yeast library to screen for proteins interacting with CL1. First, the full-length CDS of *CL1* was inserted into the BD vector to construct the bait vector, pGBKT7-*CL1*. Self-activation analysis revealed that the positive control (pGADT7-T/pGBKT7-53), negative control (pGADT7-T/pGBKT7-lam), and target gene (pGADT7-T/pGBKT7-*CL1*) all showed normal growth on double-dropout medium (SD/-Leu-Trp). However, on quadruple-dropout medium (SD/-Leu-Trp-His-Ade), only the positive control exhibited normal growth, indicating that BD-*CL1* does not possess any self-activation ability, and can be used as a bait vector for subsequent library screening (Figure 6A).

First, using the yeast two-hybrid system and a double-dropout medium lacking histidine and adenine, library screening was performed. A total of 45 clones were identified on the double-dropout medium, and sequencing analysis revealed 8 nonredundant genes. Among them, the *SRS5* [50,51] and *OsMFS1* [52] appeared most frequently in the clones (4 times each). *SRS5* encodes an alpha-tubulin protein and regulates cell elongation, independent of the BR signaling pathway, resulting in dwarfism, twisted aboveground growth, right-handed leaf and stem spiraling, shortened but non-twisted roots, increased meristematic cell numbers at the stem tip, and abnormal leaf cell shapes [50,51]. The *osmfs1* mutant exhibits pollen grains lacking starch granules, irregular shapes, male gametophyte sterility, and defective embryo sac development due to severe chromosomal abnormalities during the reproductive stages [52]. Therefore, these two genes were selected for pairwise verification analysis with *CL1*. The bait protein vectors pGADT7-*SRS5* and pGADT7-*OsMFS1* were constructed and co-transformed with the prey vector pGBKT7-*CL1* into yeast cells (Figure 6B). The growth of the transformed yeast cells on the control medium (SD/LT) and selective growth medium (SD/LTHA) indicated the interaction between CL1 and SRS5 [50,51], as well as OsMFS1 [52].

## 3. Discussion

Leaf curling is a complex trait regulated by multiple genes, and even identical leaf-curling phenotypes may be caused by different genes [53,54]. Recent research suggests that leaf curling may be attributed to mutations or variations in the size and number of bubble cells or sclerenchymatous cells [27,55]. Bubble cells are located in the middle of the vascular bundles and run parallel to the leaf veins, playing an important role in regulating leaf curling in rice [28]. Sclerenchymatous cells are crucial for maintaining leaf morphology in rice. They are derived from the differentiation of mesophyll cells, and their absence leads to axial leaf curling.

In this study, the *curling leaf 1* (*cl1*) mutant with a leaf-curling phenotype was obtained from the WuYunJing 7 mutant library, through ^60^Co-γ radiation induction. The leaves of the *cl1* mutant exhibited curling behavior, starting from the seedling stage, and continued to curl until they formed a shallot-like shape. Histological analysis of the *cl1* mutant revealed that the loss of sclerenchymatous cells on the abaxial side of the leaf vascular bundles was the key factor contributing to leaf curling. The region of sclerenchymatous cells on the abaxial side was replaced by mesophyll cells, which continuously proliferated and developed. In contrast, the sclerenchymatous cells on the adaxial side were in a cluster of dead cells without the ability to divide [56]. This disrupted the growth balance between the adaxial and abaxial sides of the leaf, resulting in inward leaf curling.

By positional cloning, the gene responsible for the phenotype was identified as *LOC_Os09g23200*. Phylogenetic analysis revealed that *CL1* is most closely related to the maize *Zm-B73* gene and the rice *OsKAN2.2* gene. *Zm-B73* belongs to the KANADI gene family and controls the polarity development of maize leaves and leaf sheaths, highlighting the role of the KANADI family in monocot leaf development [57]. Furthermore, *CL1* shares high homology with the dicot *Arabidopsis* genes *AtKAN1* and *AtKAN2*, which are also involved in regulating the development of asymmetric leaves and cellular polarity [58]. These findings suggest that the KANADI1 family may represent an important class of transcription factors that are involved in regulating plant leaf morphology, with a certain degree of conservation.

Furthermore, *CL1* shares high homology with the dicot Arabidopsis genes *AtKAN1* and *AtKAN2*, which are also involved in regulating the development of asymmetric leaves and cellular polarity. The *kan1kan2* mutants exhibited a phenotype with disorganized growth, expansion of the blade in various planes, or the development of ectopic leaf-like organs. Meanwhile, the ectopic expression of a leaf margin reporter in the abaxial was observed in the *kan1kan2* double mutants. The maize *milkweed pod1* (*mwp1*) mutant exhibits adaxialized sectors in the sheath, the proximal part of the leaf. Ectopic leaf flaps develop where the adaxial and abaxial cell types are juxtaposed. These findings suggest that the KANADI1 family may represent an important class of transcription factors involved in regulating plant leaf morphology and also affect plant yield, with a certain degree of conservation.

The *CL1* gene isolated in this study shares an allelic relationship with the *SLL1* gene identified in the rice variety “Nipponbare” [59]. The *sll1* mutant showed narrow, extremely rolled, and dark green leaves, with a deficiency of sclerenchymatous cells at the abaxial side, replacing them with mesophyll cells. Aside from an altered leaf morphology, other tissues, including the seeds, anthers, and roots, displayed abnormal development in *sll1* mutants [60]. Compared with the strong allelic variant *sll1*, *cl1* displayed a relatively weak but similar phenotype in terms of leaf curl, which is consistent with the fact that the mutation of *sll1* affects the GARP domain, while that of *cl1* does not (Figure S1 in the Supplementary Materials). These phenotypic differences indicate the importance of the GARP domain to the function of the KAN1 protein, probably because of its central role in regulating gene transcription.

Previous studies on *SLL1* have primarily focused on its regulation of leaf morphology. In this study, we also emphasize its impact on rice yield. During the maturity stage, the *cl1* mutant exhibited a significantly reduced seed-setting rate and 1000-grain weight, with values that were only 25.3% and 31.9% of those in the WT, respectively. Similar results have been reported in other mutants, such as *cd1* [61] and *lrl1* [62]. The underlying reasons could be twofold. First, it is possible that the associated genes control multiple growth and development processes in rice. Second, excessive leaf curling may lead to decreased photosynthesis and imbalanced source–sink relationships, resulting in reduced yield. Microarray analysis revealed significant downregulation of yield-related genes, such as *OsPho1* [29], *PHS8* [32], *OsBMY4* [31], and *OsGLO3* [30], in the *cl1* mutant. The expression of genes involved in regulating photosynthesis, including *OsBE1* [24], *OsCPL1* [27], *OsPsbP* [26], and *OsPS1* [25], was also markedly altered in the *cl1* mutant. These findings may contribute, to some extent, to the leaf curling and decreased yield observed in the *cl1* mutant. Yeast two-hybrid assays indicated that CL1 interacts with SRS5 [50,51]. The *Srs5* mutant exhibits smaller and rounder seeds, reduced glume cell length, rightward spiral leaf growth, and abnormal leaf cell morphology. CL1 also interacts with MFS [52], and the *mfs2* mutant shows pleiotropic defects in spikelet development, affecting grain-setting rate and yield. These results suggest that *CL1* may regulate leaf curling and grain yield by modulating *Srs5* [50,51] and *MFS* [52]. However, the specific relationship and mechanisms require further investigation.

Appropriate leaf curling can enhance sunlight penetration and improve light and energy utilization efficiency [63], thereby increasing source capacity and enhancing sink strength. However, severe leaf curling can reduce the ability of individual rice plants to intercept sunlight and the productivity of rice populations. Additionally, considering that genes regulating leaf curling also play a role in controlling plant yield, it is essential to explore more “favorable” mutation sites in these genes to strike a balance between moderate leaf curling and yield regulation, thus maximizing crop productivity. Recent research has identified three beneficial alleles, *NARROW LEAF1* (*NAL1*) [64,65] (greening for photosynthesis), *SPIKE* (*SPIKELET NUMBER*) [66], and *qLSCHL4* (leaf shape and chlorophyll content) [67] that can enhance photosynthetic capacity and increase grain yield in rice. Moreover, compared to the loss-of-function alleles of *NAL1*, beneficial allele mutations in *NAL1* can weaken or eliminate the adverse effects of fine-tuned expression or protein levels. Similarly, in the future, it would be worthwhile to further explore favorable mutation sites in *CL1* and apply them in practical agricultural production.

In conclusion, this study characterizes an important regulatory factor, *CL1*, which is involved in the regulation of leaf morphology and yield. Further studies are needed to further clarify the *CL1* regulatory network, as well as possible genetic improvements in practice.

## 4. Conclusions

In summary, in this study, a mutant with a curling leaf, *cl1*, was obtained from a mutant library generated by ^60^Co-γ radiation in the WuYunJing 7 cultivar. Through a map-based cloning method, it was confirmed that the *LOC_Os09g23200* encoding MYB protein was the candidate gene for *CL1*. *CL1* exhibited a constitutive expression pattern, with the highest expression level in the leaves. A subcellular localization study showed that CL1 was a typical nuclear protein. Genes related to photosynthesis, leaf morphology, and yield showed a significant expression change in the *cl1* mutant. Via yeast two-hybrid assays, we found that CL1 could interact with the alpha-tubulin protein SRS5 and AP2/ERF protein MFS. This study indicates that *CL1* plays an important regulatory role in rice leaf development, also affecting plant yield. Further studies are needed to further clarify the *CL1* regulatory network, as well as identify possible genetic improvements in practice.

## 5. Materials and Methods

### 5.1. Plant Materials

A stable heritable leaf-curling mutant (*cl1*) was obtained from the mutagenized library of WuYunJing 7 using ^60^Co-γ radiation. The F2 mapping population was generated by crossing with Nipponbare.

### 5.2. Investigation of Agronomic Traits

Three WT rice plants and three *cl1* mutant rice plants at the mature stage were selected. Agronomic traits such as plant height, flag leaf length, flag leaf width, flag leaf angle, second leaf length, third leaf length, panicle length, effective tiller number, seed setting rate, and 1000-grain weight were investigated and recorded. During the seedling stage, the first fully expanded leaf and the third leaf of the *cl1* mutant, as well as leaves of the same age from the WT (control), were selected. In the mature stage, flag leaves of both the mutant and WT plants were collected. Following the experimental protocol described by Feng et al. (2015) [68], paraffin sections were prepared for the three leaf ages of both the mutant and WT plants. Observation and photography were conducted under a light microscope. Frontal and transverse sections of the flag leaf were photographed under a dissecting microscope, and the rice panicles and grains were photographed using a camera.

### 5.3. Determination of Photosynthetic Efficiency and Photosynthetic Pigment

At a high temperature and under strong sunlight, three WT and three *cl1* mutant rice plants at the mature stage were selected. A handheld photosynthesis measurement instrument was used to measure the net photosynthetic rate of the flag leaf, the second leaf from the top, and the third leaf from the top of each plant, following the detailed experimental protocol described by Murat et al. (2021) [69]. Fresh leaves from the same position and orientation of three WT and *cl1* mutant plants at the seedling stage were selected. Approximately 0.2 g of the leaves was weighed and thoroughly ground with a suitable amount of alcohol, a small amount of SiO_2_, and CaCO₃. The thoroughly ground mixture was filtered into a brown volumetric flask and made up to 25 mL. A blank control was prepared using 95% alcohol. The absorbances at 665 nm, 649 nm, and 470 nm were measured using an enzyme-linked immunosorbent assay (ELISA) reader. The obtained OD values were then used in the following formulas to calculate the content (mg/g) of Chla and Chlb:

Ca = (13.95 × OD665 − 6.88 × OD649) × V/(W × 1000)(1)

Cb = (24.96 × OD649 − 7.32 × OD665) × V/(W × 1000)(2)

Cchl = Ca + Cb(3)

(V: final volume after making up the solution, W: weight of the measured leaves.)

### 5.4. Map-Based Cloning of CL1

Using a map-based cloning strategy, we isolated the *CL1* gene, following a detailed experimental protocol described by Chen et al. (2019) [70]. A total of 480 F2 individuals derived from the cross between *cl1* and Nipponbare were selected as the mapping population. SSR markers that were uniformly distributed across the 12 chromosomes were utilized to screen for polymorphisms in the mutated gene pool (10 randomly chosen *cl1* mutant plants from the F2 population, exhibiting leaf curling), wild-type, and Nipponbare. Recombination frequencies were calculated, and genetic map distances between the linked markers and the mutated gene were determined. Genomic resequencing was performed on individual wild-type and Nipponbare plants to identify the differential indels (insertions or deletions of nucleotides). Based on the preliminary mapping results and resequencing data, primer software was used to design primers for the corresponding positions on the chromosome harboring the mutated gene. By calculating the genetic map distance between the indel site and the mutated gene using linked markers, the candidate region for the mutated gene was gradually narrowed down through the use of new marker primers.

### 5.5. The Expression Pattern and Subcellular Localization of *CL1* for RT–PCR Analysis

RNA was extracted from the roots, stems, leaves, panicles, anthers, and wild-type leaf samples at the various developmental stages of rice. The extracted RNA was reverse-transcribed into cDNA and specific primers for *CL1* (Table S2 in the Supplementary Materials) were designed for RT–PCR analysis, with *OsACTIN1* serving as the internal control. Gene-specific primers were used to amplify the *CL1* coding sequence (Table S2), which was then cloned into a separate 1300s-GFP vector. The resulting recombinant vector was transformed into rice protoplasts using polyethylene glycol-mediated transfection. Rice protoplasts were grown in the dark at 28 °C for 12–16 h. The fluorescence of the protoplasts was observed using a Nikon C2-ER confocal laser scanning microscope; the excitation wavelength of green fluorescent protein (GFP) is 488 nm and the emission wavelength is 510 nm [71].

### 5.6. Transcriptome Analysis

The WT and *cl1* mutant leaves at the seedling stage were selected for transcriptome analysis; three randomly selected plants represented a biological duplication, with a total of three replicates. The cDNA libraries of the rice leaf curl *cl1* mutant and the WT cultivar WuYunJing 7 were constructed and subjected to high-throughput sequencing by the Omicshare Biotech Company. The read count data obtained from the gene expression analysis were analyzed using DESeq2 software to identify differentially expressed genes. Genes with a false discovery rate (FDR) < 0.05 and |log_2_FC| > 1 were considered to be significantly differentially expressed genes.

The differentially expressed genes were mapped to various terms in the Gene Ontology (GO) database (http://www.geneontology.org/, accessed on 20 January 2023) to determine the number of differentially expressed genes associated with each term. This allowed the generation of a list of differentially expressed genes and the statistical analysis of the number of genes associated with each GO term. The hypergeometric test was then applied to identify significantly enriched GO terms among the differentially expressed genes, compared to the background.

To validate the reliability of the transcriptomic sequencing results, ten differentially expressed genes were randomly selected for qRT–PCR verification. Leaf cDNA samples from both the WT and the *cl1* mutant were used as templates for PCR amplification.

### 5.7. Screening of *CL1* Interacting Proteins

We used the yeast two-hybrid method to screen the CL1 interacted proteins, following the detailed experimental protocol described by Fang et al. (2022) [72]. The BD vector containing the *CL1* gene and the empty AD vector were co-transformed into yeast-competent cells. The transformed cells were plated on a selective medium lacking leucine and tryptophan (double-dropout medium), as well as on a medium lacking leucine, tryptophan, histidine, and adenine (quadruple-dropout medium). The plates were inverted and incubated at 30 °C for 3 days to observe the growth of yeast colonies and determine if there was self-activation.

The AD vector containing the target gene and the BD vector containing the *CL1* gene were separately co-transformed into yeast-competent cells. The cells were incubated at 30 °C for 3 days until single colonies were formed. Single colonies were then picked out and inoculated into liquid media lacking leucine and tryptophan (double-dropout liquid medium) and lacking leucine, tryptophan, histidine, and adenine (quadruple-dropout liquid medium). The cultures were incubated at 30 °C for 2–3 days. Aliquots of 5 µL of each culture were then spotted onto double-dropout and quadruple-dropout solid media for further analysis.

## Data Availability

All relevant data are within the paper.

## Supplementary Materials

The following supporting information can be downloaded at: https://www.mdpi.com/article/10.3390/plants12173127/s1, Table S1: Genetic prediction within the localization interval; Table S2: The primer pairs used in this study. Figure S1: The mutation locus of *sll1* and *cl1*.
