# Supplementary material for: Curling Leaf 1, Encoding a MYB-Domain Protein, Regulates Leaf Morphology and Affects Plant Yield in Rice
Source: Plants (Basel). 2023 Aug 31;12(17):3127. doi: 10.3390/plants12173127 (PMC10490398; doi:10.3390/plants12173127)

Supplementary Table S1 Genetic prediction within the localization interval

Locus name	Gene annotation
Os09g23084	endoglucanase, putative, expressed
Os09g23100	retrotransposon protein, putative, Ty1-copia subclass, expressed
Os09g23110	transporter family protein, putative, expressed
Os09g23120	retrotransposon protein, putative, Ty3-gypsy subclass, expressed
Os09g23130	retrotransposon protein, putative, unclassified, expressed
Os09g23140	endonuclease/exonuclease/phosphatase family domain containing protein, expressed
Os09g23150	monoglyceride lipase, putative, expressed
Os09g23160	annexin, putative, expressed
Os09g23170	expressed protein
Os09g23180	expressed protein
Os09g23190	transposon protein, putative, CACTA, En/Spm sub-class, expressed
Os09g23200	KANADI1, putative, expressed

Supplementary Table S2 The Primer Pairs used in this study

Primer name	Primer Sequence(5-3')
9-6F	ATAAACTTCCAACTTTTCCGTC
9-6R	TAGAATATCCCGTGATTGTGGC
9-7F	GCTGGTTCACGCCGCCTTTGGG
9-7R	ATTGCGCCGACGTTTTGGACAT
cl1-1F	TCAGAATTCAGTTAGTCCCCAAT
cl1-1R	AACACGCACAAACGAAAAAAAAT
cl1-2F	TTCAGTTAGTCCCCAATTGATCC
cl1-2R	AATGCGAGCTAATGAATTTCTGC
cl1qRT-F	GATACCTATCTACAACGGCCC
cl1qRT-R	GTACGAGCTGTAGAATCCCATC
cl1GFP-F	cctcgcccttgctcaccatggtaccATCATGATCTG CACCGTGCCagtc
cl1GFP-R	ctcgagctttcgcgagctcggtaccATGGCAATGG TGCGGGAGctcgag
OsBE1qRT-F	AGGTATTCAAGTTGGTCCGAAT
OsBE1qRT-R	CAAATCAGAGACGTCGAACATC
OsPS1qRT-F	GGGTGTAGAAGCTGATAGGATC
OsPS1qRT-R	ACACAAGAGAGAGGTACGGTAT
OsPsbPqRT-F	CGTCGAAATCTCTATCTCAGCT

OsPsbPqRT-R	CGACCTCTCTCCTTAACTTGTT
OsCPL1qRT-F	GACCATCACGTTCAAGAACAAC
OsCPL1qRT-R	GTACTCCTCCTGCGATATCTTG
SLL1qRT-F	CAGCTCGCAGGTGTCCAA
SLL1qRT-R	CCTCCCTAGAGTGAACTCGAGACT
NAL7qRT-F	CAAGAACATCACCGGCAAGA
NAL7qRT-R	CGATTTGATCAAGGACCATGCT
SRL1qRT-F	CCCAATTCCTTGGTGACAAGT
SRL1qRT-R	TTGCATGAAGAAGACGATGCT
OsPho1qRT-F	AATGTGAAGCAAGCGTATTACC
OsPho1qRT-R	CTGTGTCCAAGTTGTTGTAGTG
OsGLO3qRT-F	CTGGGAGCTTCAGGAGTATTTA
OsGLO3qRT-R	AGCATCTCAAATGGACTAGCAT
OsBMY4qRT-F	CAGTCACTATACCACTTCCGAA
OsBMY4qRT-R	CGACTTGAATCTCGACTATGGT
PHS8qRT-F	ACAGTATCAACTTTGTGTGTGC
PHS8qRT-R	TCAATCTTTTGACCGACAAACC
TGW3qRT-F	AAACACTCGTCTACCTAATGGG
TGW3qRT-R	CTCAGGAAGAAAAACTGCGAAA
TGW6qRT-F	CACAACGAGAATGTTCAAGACC
TGW6qRT-R	CACTTGTTTTTCGTGTAGCTGG
OsCBSX3qRT-F	AAGGGAGCAAAGATCAATGGTA
OsCBSX3qRT-R	AGGGTTCTCGTCGATCTCTAG
OsDi9-3qRT-F	GAGATGGAGATGGAGGATGATG
OsDi9-3qRT-R	AATCTTTTCGAAGCAGATGGGA
RSUS1qRT-F	TCAGCGTATCGAGGAGAAATAC
RSUS1qRT-R	AATGCTCTCCTCTTTCAGAGTC
U2AFqRT-F	GTAGAGCATCACTGGGTAGTAC
U2AFqRT-R	CCACATTAATCGGTTACCCCTA
Srs5qRT-F	TGACGAGTACTAGAGAGGTTCA

Srs5qRT-R	CAAAACCAGACACACAAACTGA
OsMFS1qRT-F	GTAGAGCATCACTGGGTAGTAC
OsMFS1qRT-R	CCACATTAATCGGTTACCCCTA
ACTIN-F	CGGGAAATTGTGAGGGACAT
ACTIN-R	AGGAAGGCTGGAAGAGGACC

Supplementary Figure S1: The mutation locus of *sll1* and *cl1*.

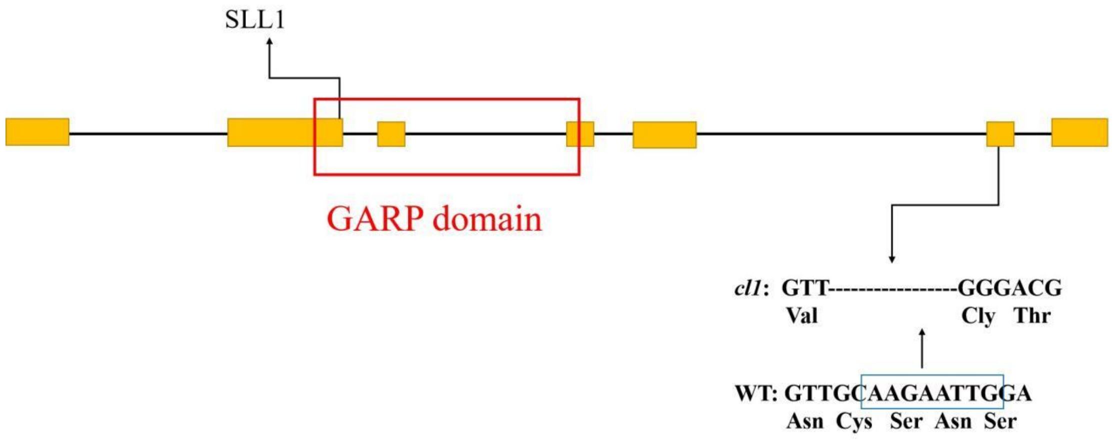

Supplement: Supplementary file 1 [file plants-12-03127-s001.zip › plants-2586801-supplementary.pdf]
